# Expression of pendrin in benign and malignant human thyroid tissues

**DOI:** 10.1038/sj.bjc.6602628

**Published:** 2005-06-07

**Authors:** J Skubis-Zegadło, A Nikodemska, E Przytuła, M Mikula, K Bardadin, J Ostrowski, B E Wenzel, B Czarnocka

**Affiliations:** 1 Department of Biochemistry, Medical Centre for Postgraduate Education, Marymoncka 99, 01-813 Warsaw, Poland; 2 Department of Pathology, Medical Centre for Postgraduate Education, Cegłowska 80, 01-809 Warsaw, Poland; 3 Department of Gastroenterology, Medical Centre for Postgraduate Education, Oncology Centre, M. Skłodowska - Curie Memorial Institute, Roentgena 5, 02-781 Warsaw, Poland; 4 Cell & Immunobiology Laboratory, Department of Medicine I, Medical University, Ratzeburger Allee 160, D-23538 Lübeck, Germany

**Keywords:** pendrin, quantitative RT–PCR, Western blot, immunohistochemistry (IHC), papillary thyroid carcinoma (PTC), follicular thyroid carcinoma (FTC)

## Abstract

The Pendred syndrome gene (PDS) encodes a transmembrane protein, pendrin, which is expressed in follicular thyroid cells and participates in the apical iodide transport. Pendrin expression has been studied in various thyroid neoplasms by means of immunohistochemistry (IHC), Western blot and RT–quantitative real-time PCR. The expression was related to the functional activity of the thyroid tissue. Follicular cells of normal, nodular goitre and Graves' disease tissues express pendrin at the apical pole of the thyrocytes. In follicular adenomas, pendrin was detected in cell membranes and cytoplasm simultaneously in 10 out of 15 cases. Pendrin protein was detected in 73.3 and 76.7% of the follicular (FTC) and papillary (PTC) thyroid carcinomas, respectively, where pendrin was solely localised inside the cytoplasm. An extensive intracellular immunostaining of pendrin was observed in six out of 11 (54.5%) of positive FTCs and 19 out of 23 (82%) of PTCs. Focal reactivity was detected in one follicular- and three papillary carcinomas, whereas pendrin protein was absent in three of 15 FTC and four of 30 PTC; mRNA of pendrin was detected in 92.4% of thyroid tumours. The relative mRNA expression of pendrin was lower in cancers than in normal thyroid tissues (*P*<0.001). The pendrin protein level was found to parallel its mRNA expression, which was not, however, related to the tumour size and tumour stage. In conclusion, pendrin is expressed in the majority of differentiated thyroid tumours with high individual variability but its targeting to the apical cell membrane is affected.

The iodide metabolism and thyroid hormone synthesis in the thyroid gland is controlled by specialised proteins. The iodine pathway comprises several steps that involve (1) the transportation of iodide from the blood stream to the intracellular compartment by NIS localised in the basal membranes of the follicular cells (Dai *et al*, 1996; Smanik *et al*, 1996) and (2) the intracellular iodide transportation to the follicular lumen, where iodide accumulation, oxidation, and organification – key steps of hormonogenesis – normally occur (Taurog, 2000). The basolateral iodide transport as well as the NIS, a protein that catalyses this process are well characterised (Dai *et al*, 1996; Smanik *et al*, 1996; Dohan *et al*, 2003). However, the apical translocation of iodide across membranes and the protein/s involved are still a matter of debate in the pathophysiology of the thyroid gland (Everett *et al*, 1997; Rodriguez *et al*, 2002). One of the iodide transporters located at the apical pole of the thyrocytes is pendrin encoded by the PDS gene (Pendred syndrome gene), a member of the SCL26A gene family (Everett *et al*, 1997; Royaux *et al*, 2000). Pendrin is a highly hydrophobic transmembrane protein composed of 780 amino acids with 12 trans-membrane domains (Everett *et al*, 1997; Royaux *et al*, 2000). In the thyroid, pendrin is localised in the apical membrane of follicular cells facing the colloid lumen. It was demonstrated to function as an iodide transporter (Taylor *et al*, 2002; Yoshida *et al*, 2002). Electrophysiological studies suggest the existence of two apical iodide channels (Golstein *et al*, 1992), and as recently shown, pendrin mediating vectorial iodide transport corresponds to one of these channels (Gillam *et al*, 2004). The pendrin gene is not under the control of TSH but it is upregulated by thyroglobulin (Royaux *et al*, 2000). It has been postulated that the loss of the transport capability of pendrin may be responsible for the partial defect of organification, observed in patients with the Pendred syndrome (Reardon *et al*, 1999; Scott *et al*, 1999).

The role of pendrin in the pathophysiology of the thyroid is not fully understood. Recently, the pendrin gene- and protein expression have been studied in different thyroid diseases (Bidart *et al*, 2000; Arturi *et al*, 2001; Lacroix *et al*, 2001; Porra *et al*, 2002; Kondo *et al*, 2003). It was found that the PDS gene transcript level in thyroid tumours was slightly (Porra *et al*, 2002) or dramatically (Bidart *et al*, 2000; Lacroix *et al*, 2001) reduced or even absent (Arturi *et al*, 2001; Mian *et al*, 2001), compared to normal thyroid tissues. Moreover, in thyroid tumours, an aberrant hypermetylation of the PDS gene was observed (Xing *et al*, 2003). In hypo- and hyperfunctioning adenomas, the PDS gene expression and protein levels were within the normal range, while they were increased in toxic adenomas (Bidart *et al*, 2000; Arturi *et al*, 2001). Thyroid cancers have different defects of the iodide metabolism, which may occur at each step of the iodide pathway, thus including defects of the iodide transporters (Hoang-Vu *et al*, 1992).

Our study has focused on the immunological and immunohistochemical analysis of a series of normal, benign and malignant thyroid tissues with pendrin-specific antibodies, as well as on the quantification of the pendrin gene expression. The results indicate that pendrin is easily detected in most differentiated thyroid carcinomas on both mRNA and protein levels. Malignant transformation, however, interferes with proper pendrin trafficking to the apical plasma membrane in tumour cells.

## MATERIAL AND METHODS

### Thyroid tissues

A total of 155 thyroid tissue samples were provided by the Centre of Oncology, Department of Surgery as well as by the Pathology Department of CMKP. After histopathological examination, tissues were classified according to the WHO recommendations and selected. Each tumour was scored based on the TNM (tumour-node-metastasis) classification (Table 2, Sobin and Fleming, 1997). The samples chosen for the study represented a wide range of thyroid pathologies such as Graves' thyroid tissue (GD), *n*=10; nodular goitre (NG), *n*=28; follicular adenoma (FA), *n*=15; follicular carcinoma (FTC), *n*=15; papillary carcinoma (PTC), *n*=30; medullary carcinoma (MTC), *n*=8 and anaplastic carcinoma (ATC), *n*=2, which were compared to the normal thyroid (NT), *n*=47. Tissues obtained after surgery were immediately frozen and stored in liquid nitrogen until analysis. For Western blot and RNA isolation, the cancerous tissue was separated from the surrounding normal thyroid fragments by expert evaluation of the pathologist.

The local human study ethical committees approved this study and all patients gave their informed consent.

### Antibodies

Two peptides, numbered 1, M-A-A-P-G-G-R-S-E-P-P-Q-L-P-E, corresponding to the first 15 amino acids (1–15) and peptide 5, L-D-V-Q-D-E-A-M-R-T-L-A-S, corresponding to the last 13 amino acids (768–780) of the human pendrin sequence (Everett *et al*, 1997), were synthesised by the conventional solid phase method, and used to prepare antipeptide antisera in rabbits. Briefly, the peptide was conjugated to mariculture keyhole limpet haemocyanin (1 mg peptide/1 mg haemocyanin) through a terminal cysteine residue (added at the C-terminal end in peptide 1 or to the N-terminal end in peptide 5) with sulpho-SMCC (sulphosuccinimidyl 4-*N*-maleimidomethyl cyclohexane-1-carboxylate (Pierce Chemical Co, Rockford, IL, USA) as a crosslinker according to the manufacturer's instruction. This peptide conjugate was then used for the immunisation procedure (two New Zealand White female rabbits were injected) according to the standard schedule. Sera were tested for their antibody titre against the peptide by ELISA. Appropriate controls with preimmune sera at the same dilutions were used and controls for nonspecific binding were run parallely.

Then site-specific antipeptide antibodies were purified by affinity chromatography on peptide-coupled columns (sulpho-link gel, Pierce Chemical Co.). Both antibodies, to N-, and C-peptides, produced identical results in immunological studies (Table 1).

### RT–real-time PCR analysis

Quantitative RT–real-time PCR analysis for *pendrin* and *β-actin* were performed using the GeneAmp 5700 Sequence Detection System (PE Applied Biosystems, Foster City, CA, USA). Total RNA was isolated from frozen thyroid tissues using RNeasy Mini Kit (QIAGEN, Hilden, Germany). The RNA concentration and purity were determined by absorbance measurements at 260 nm and by determining the A 260/A 280 ratio. In all, 1 *μ*g of total RNA was reverse-transcribed using SuperScript II RT (GIBCO-BRL, Gaithersburg, MD, USA) and random hexamers in 20 *μ*l of volume as per the manufacturer's protocol. The cDNAs were then diluted 1 : 25 in 10 mM Tris-HCl pH 8.0. Oligonucleotide primers and TaqMan probe for *PDS* gene (TaqMan Assays-on-demand gene expression, Id: Hs00166504_m1) and *β-actin* reference gene (Id: Hs99999903_m1) products were purchased from PE Applied Biosystems. DNA polymerase in TaqMAN universal PCR Master MIX was activated by incubation for 10 min at 95°C. PCR amplification was carried out at 40 cycles, consisting of 15 s of denaturation at 95°C and hybridisation of primers and a probe for 1 min at 60°C in a 96-well reaction plate; all analyses were performed in duplicate and the mean was calculated. The normal control thyroid tissue (pool of tissues) was used for the construction of a standard curve for both PDS and *β*-actin. Four differently diluted cDNAs (200 ng, 50 ng, 12.5 ng, 3.125 ng in 25 *μ*l well^−1^) of the control thyroid were used to construct a standard curve. The target quantity of pendrin was determined from the standard curve and divided by the target quantity of *β*-actin RNA for normalisation. The relative values of pendrin were expressed as *n*-fold differences from the mean values of the normal thyroid. Quantitative real-time PCR data analysis was carried out using Microsoft® Excel® based software application Q-Gene (Bernard and Wittwer, 2002; Muller *et al*, 2002).

PCR amplification efficiencies of the PDS gene and *β*-actin gene were as follow: *E*_pds_=1.95 and *E*_*β*-actin_=1.96.

### SDS–PAGE and Western blotting

Tissue samples were homogenised in an ice-cold buffer (250 mM sucrose, 20 mM Tris-HCl, pH 7.4, 1 mM EDTA) containing a cocktail of protease inhibitors (Roche diagnostics GmbH, Mannheim, Germany). Homogenates were centrifuged at 1000 *g* for 15 min at 4°C, then at 100 000 *g* for 60 min at 4°C. The resulting pellets containing particulate fractions were recovered, resuspended in 20 mM Tris-HCl, pH 7.4 with 1 *μ*g ml^−1^ PMSF (phenyl-methyl-sulphonyl fluoride) and kept at −80°C. The protein concentrations were evaluated by the BCA (bicinchoninic acid) protein assay reagent (Pierce Chemical Co.).

In total, 30 *μ*g of crude membrane proteins were mixed with a protein buffer (0.25 mM Tris-HCl, pH 6.8; 20% glycerol; 4% SDS and 0.1% bromophenol blue) and incubated with 0.125 M dithiothreitol for 30 min at 37°C. Each sample was loaded into individual wells and electrophoresed through a 9% acrylamide, using SDS–PAGE method. Proteins were electrotransferred to Immuno-blot PVDF membranes (Bio-Rad Laboratories, Hercules, CA, USA), which were then saturated with 5% powdered milk in PBS-Tween (PBS-T). Western blotting experiments were subsequently carried out by incubating the blotted membranes for 2 h with anti-pendrin antibody at 1 *μ*g ml^−1^ concentration at room temperature. After three washings in PBS-T for 10 min, the membranes were incubated with affinity-purified anti-rabbit antibody labelled with horseradish peroxidase (Jackson ImmunoResearch Laboratories, West Grove, PA, USA) for 1 h at room temperature under shaking. After extensive washing, membranes were developed with a chemiluminescent peroxidase substrate SuperSignal® West Pico (Pierce Chemical Co.) for 5 min at room temperature, then exposed for 15 min to Biomax film MS (Eastman Kodak Co. Rochester, NY, USA) according to the manufacturer's instructions. The free peptide precipitation of primary antibody as well as secondary antibody controls were included in the series of Western blots.

Western blot signals were scanned and quantified using KODAK 1D Image Analysis Software (Eastman Kodak Co. Rochester, NY, USA).

### Immunohistochemistry (IHC)

Tissue sections (3 *μ*m) mounted on silane-coated glass slides were deparaffinised in xylene, rehydrated via graded ethanols to water ratios. After antigen heat retrieval (water bath, 96°C in 10 mM citric buffer, pH 6.0, for 20 min) and endogenous peroxidase quenching (3% H_2_O_2_ solution for 5 min), slides were incubated with site-directed anti-pendrin antibodies at a concentration of 0.25 *μ*g ml^−1^ overnight at 4°C. Then, they were washed and incubated with an LSAB 2 kit (DakoCytomation, DAKO Corp., Carpinteria, CA, USA), developed with DAB chromogene, counterstained with Mayer's haematoxylin in agreement with the manufacturer's protocols. The specificity of the immunostaining was checked by omission of single steps in the protocol (negative control), by replacement of the primary antibody with preimmune serum and peptide competition tests. Immunoreactivity was competitively inhibited by addition of 10 *μ*g of the corresponding synthetic peptides used to generate Abs.

The positive immunostaining was evaluated using a semiquantitative score: 0, negative; ++, moderate; +++, intense. Two pathologists (EP and KB) independently evaluated immunohistochemical staining on randomly numbered slides.

The FITC-labelled anti-rabbit secondary antibodies (Jackson ImmunoResearch Laboratories) were used for confocal microscopy. Pictures were acquired with the Confocal Laser Scanning Microscope (Olympus Optical Europe, Hamburg), equipped with the Fluoview ver.2.1.39 software program (Olympus). The Photoshop software (v6; Adobe System Inc.) was used to edit viewing.

### Statistical analysis

Intergroup differences in the mRNA values were analysed using the Mann–Whitney's *U-*test. The differences were considered statistically significant at *P*<0.05.

## RESULTS

### RT–PCR and Western blot analysis

**Figure 1:**
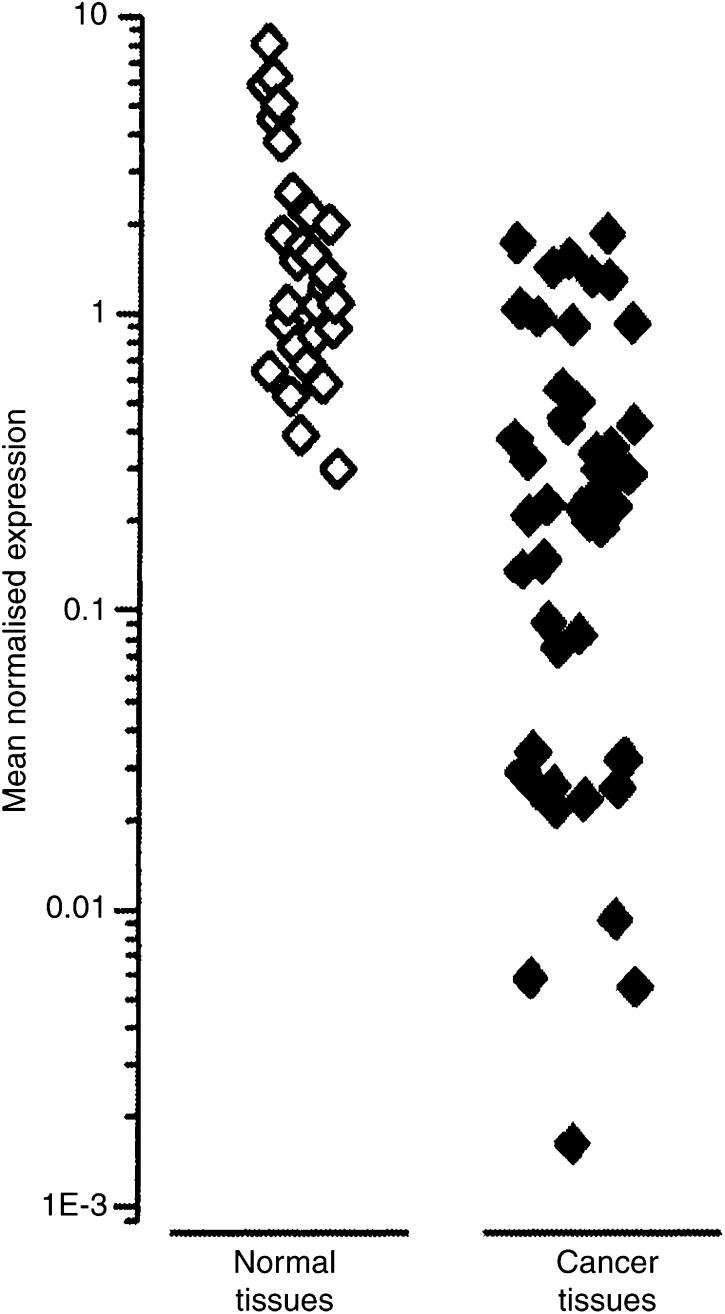
Relative level of pendrin mRNA expression in differentiated thyroid carcinomas compared to the paired normal tissues.

The expression of pendrin was determined in a series of normal and pathological thyroid tissues. Pendrin mRNA was detected by RT–real-time PCR in 49 out of 53 (92.4%) carcinomas, in the range of 0.006–1.86 relative levels. In the normal paired tissues, relative pendrin mRNA level ranged from 0.3 to 8.06 and was significantly higher compared to cancer tissue (*P*<0.001) (Figure 1). According to pendrin mRNA levels, the analysed thyroid carcinomas could be divided into four subgroups. The first two groups include cases with negative (4 out of 53; 7.5%) and low pendrin mRNA expression, (12 out of 53; 22.6 %). The next group comprises cases with mRNA levels close to the average value of MNE for all 53 analysed tissues (27 out of 53, 51%; MNE range from 0.1 to 0.6). In the fourth group of tissues, the relative pendrin mRNA levels in cancer tissues were of the same extent as mRNA levels detected in normal paired thyroid tissues (10 out of 53, 18.9%; MNE range from 0.9 to 2), Figure 1. In the group of tumours with very low mRNA expression, the differences between cancer and normal paired tissues were lower by a factor of 25–300. In the remaining cancer tissues, the PDS transcript levels were lower by a factor of 1.5–10.

**Figure 2:**
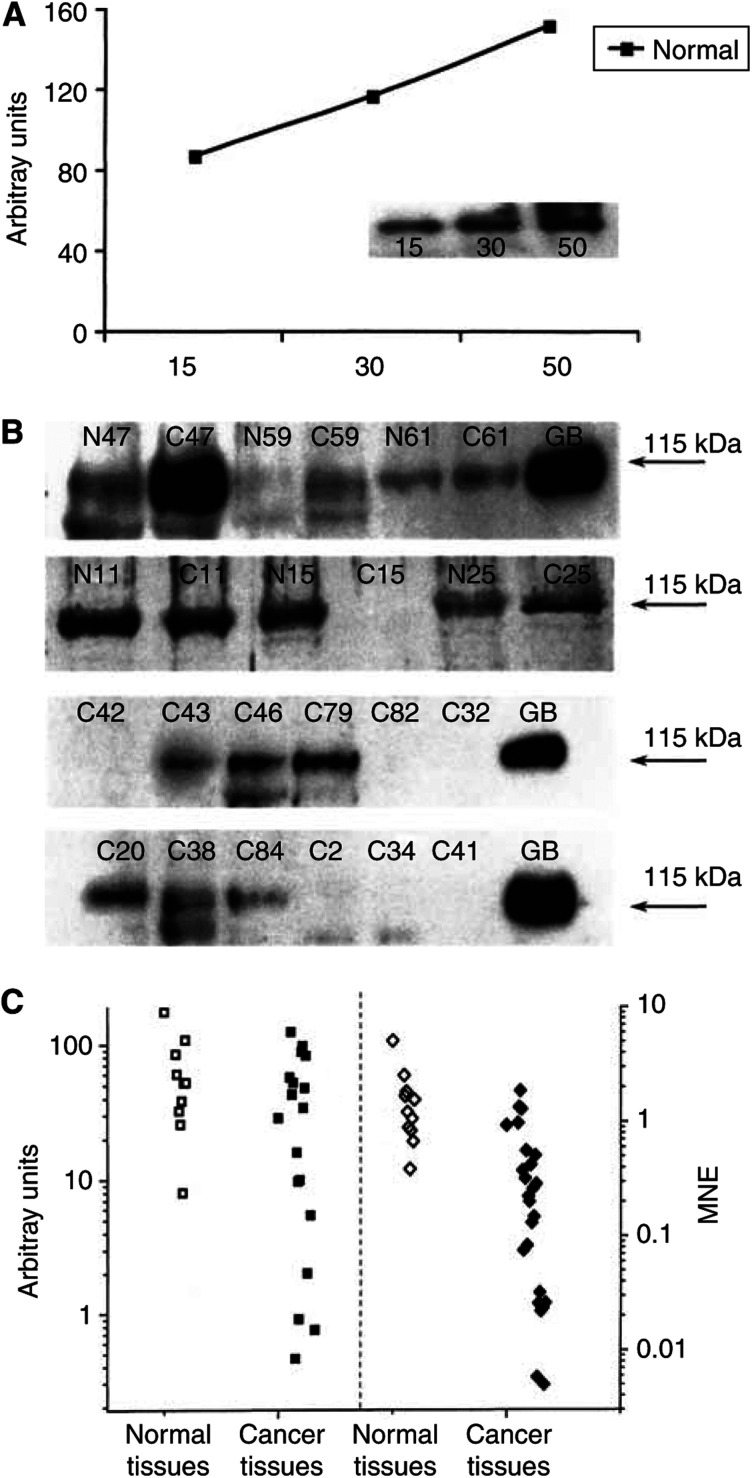
Pendrin expression in thyroid tissues. (**A**) Semiquantitative immunodetection of pendrin protein in human thyroid tissues by Western blotting. Increasing amounts of crude membrane preparation from the pool of normal thyroid tissues was used for Western blot procedure. Arbitrary units were assigned to each protein band following scanning. West Pico signals were scanned and semiquantified as described in Material and Methods. The relative amount of pendrin, expressed as arbitrary units, is plotted as a function of the micrograms of the protein loaded. (**B**) Western blotting analysis of pendrin protein expression in human thyroid tissues. Protein (30 *μ*g) from particulate fractions prepared from normal thyroid tissues (N), thyroid cancer tissues (C) and Graves' disease tissues (GD) were submitted to Western blot procedure using affinity-purified pendrin antibodies (1 *μ*g ml^−1^) and West Pico substrate. Each lane was loaded with an equal amount of the crude membrane protein (30 *μ*g) extracted from cancer and paired normal tissues. Arrows show the pendrin bands (kDa). (**C**) Comparative analysis of pendrin protein and PDS gene expression in a series of 35 thyroid cancer (C) and normal paired (N) tissues. Relative pendrin mRNA level expressed as MNE values (depicted in the right panel); pendrin protein level in those tissues expressed as arbitrary units of the luminance (left panel).

The expression of pendrin was confirmed at the protein level by Western blot with site-specific pendrin antibodies. Pendrin protein was detected in 35 out of 35 normal thyroid tissues, and in 26 out of 35 tumours (Western blotting analysis was performed for only 35 tissues because the weight of 18 cancer tissues was very small and therefore these tissues were not available for the Western blot studies.). The intensity of the pendrin band was proportional to the concentration of the membrane protein loaded (Figure 2A). Affinity-purified polyclonal anti-pendrin antibodies under denaturating conditions revealed a single polypeptide band of 115 kDa in both normal and cancer tissues (Figure 2A and B). Western blot signals obtained from six representative paired samples and 12 cancer samples of 35 are shown in Figure 2B. Using identical amounts of protein allowed measurement of the relative quantity of the pendrin with respect to intensity and size of pendrin bands. The intensity of the immunostaining of pendrin bands was closely related to the mRNA level assessed by quantitative real-time RT–PCR. Values obtained by semiquantification of pendrin protein expressed as arbitrary units and quantification of pendrin transcript signals corresponding to the tumours and paired normal tissues are shown in Figure 2C. Only cancer tissues with negative and low mRNA levels were negative in Western blot with both antibodies. In the remaining cancer samples, the pendrin band intensity varied from very weak to strong (Figure 2B).

The average level of pendrin protein as estimated from Western blot analysis was twice as low in cancer tissues as in paired normal tissues and expressed in arbitrary units was 27.9±36.3 *vs* 63.5±48.4 (mean±s.e.m.) for the paired normal thyroid.

### Immunochistochemistry for pendrin

**Figure 3:**
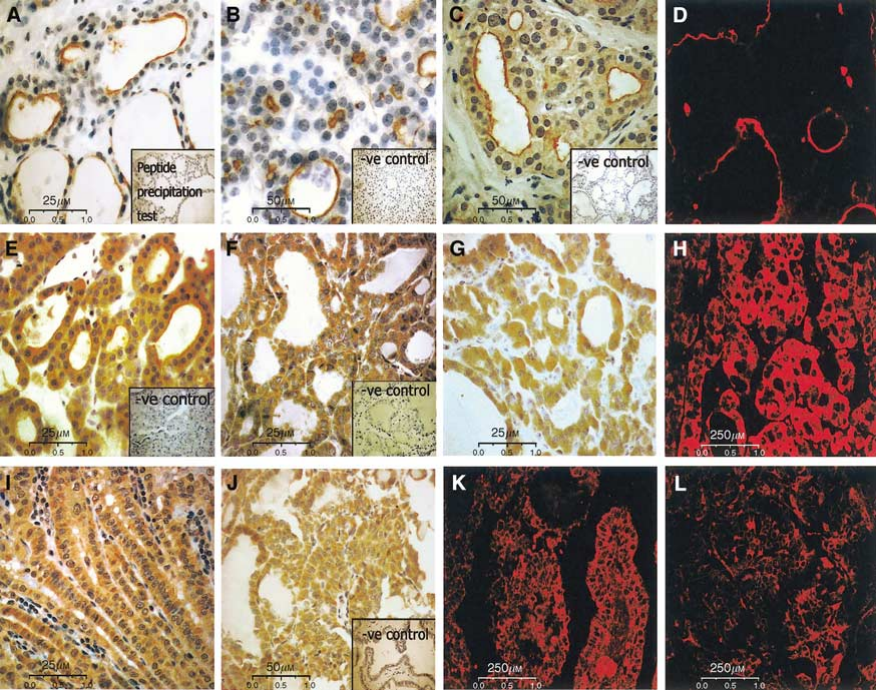
Immunohistochemical analysis of expression and cellular distribution of pendrin in normal and pathological thyroid tissues. (**A**) Normal thyroid tissue; the apical staining is heterogeneous inside and between follicles (original magnification × 400; inset – peptide competition test control); (**B**) immunostaining for pendrin in NG. The staining is restricted to the small proliferating follicles (original magnification × 200; inset – peptide competition test control); (**C**) Graves' tissue with distinct apical staining pattern, especially observed in small follicles (original magnification × 200; inset – peptide competition test control); (**D**) immunostaining of pendrin in Graves' tissue by confocal microscopy, FITC-labelled anti-rabbit secondary antibody were used. (**E**) Follicular adenoma; immunostaining shift to the cytoplasm is observed (original magnification × 400; inset – peptide competition test control); (**F**) Follicular carcinoma; strong intracellular staining (original magnification × 400); (**G**) Follicular carcinoma; moderate intracytoplasmic stain (original magnification × 400); (**H**) immunofluorescence of FTC with strong intracytoplasmic pendrin expression (original magnification × 40); (**I**) Papillary carcinoma; strong intracellular staining (original magnification × 400; inset – peptide precipitation test control); (**J**) Papillary thyroid carcinoma; moderate cytoplasmic staining (original magnification × 200); (**K**) immunofluorescence of PTC with strong pendrin expression (original magnification × 40); (**L**) immunofluorescence of PTC with moderate pendrin expression (original magnification × 40).

The result of immunohistochemical analyses of the pendrin in 155 thyroid tissues are summarised in Table 1 and are shown in Figure 3. In the normal thyroid, GD and NG tissues, the pendrin staining was limited to the apical cell membrane (Figure 3A). One of 15 follicular adenomas showed exclusively membranous pendrin expression. In 10 of 15 (66.7%) adenomas, the expression of pendrin protein was detected simultaneously in the cytoplasm and at the apical cell membrane with extensive and moderate immunostaining detected in half of all cases. Four of 15 adenomas (26.7%) expressed pendrin protein in the cytoplasm only with strong immunoreactivity detected in three cases. In a series of differentiated thyroid carcinomas, 73.3% (11 out of 15) of FTC and 76.6% (23 out of 30) of PTC clearly showed pendrin protein expression. Out of 11 follicular carcinomas, 10 (91%) expressed pendrin protein exclusively in the cytoplasm, and one both in the cytoplasm and at the apical cell membrane. In six of 10 FTC, the immunostaining for pendrin was intensive and homogenously distributed through the tumoral area, and in the remaining four cases intermediate reactivity was observed. Three (20%) follicular carcinomas were negative and one exhibited focal pendrin immunostaining. Of 30 papillary carcinomas, 23 showed pendrin protein expression. In all positive tumours, the pendrin protein was localised exclusively in the cytoplasm. Out of these 23 cases, 19 PTC showed an intensive homogenous pendrin expression, whereas in the remaining four cases moderate immunostaining was found. Four (13.3%) papillary carcinomas were negative for pendrin staining, and three showed focal reactivity (Table 1). Pendrin protein immunodetection on paraffin-embedded sections is shown in Figure 3. Affinity-purified anti-pendrin antibodies specifically labelled pendrin located at the apical plasma membranes of follicular cells of the normal thyroid (Figure 3A), NG (Figure 3B) and GD (Figure 3C) tissues. In the normal thyroid, the apical labelling was not uniform in both follicles and epithelial cells. These stained cells likely correspond to functionally active cells and follicles. In NGs (Figure 3B) and GD thyroids (Figure 3C), immunostaining varied from one follicle to another and was related to the size of the follicles (Figure 3B and C). The majority of positive cells were in small follicles and in follicles with proliferative activity, whereas in large follicles the proportion of positive cells was decreased (Figure 3B and C). In follicular adenomas, pendrin-positive immunostaining was observed at both, the apical plasma membranes and in the cytoplasm of 14 out of 15 cases (Figure 3E). In FTC and PTC, the pendrin protein immunostaining of variable intensity was uniformly distributed throughout the tumoral area and generally located in the cytoplasm and probably membrane-associated. Figure 3F+I shows cases of and PTCs with an extensive intracellular pendrin expression, and Figure 3G+J illustrates follicular and papillary carcinomas with moderate pendrin expression. The pattern of reactivity was confirmed for all tissues analysed by confocal fluorescent microscopy (Figure 3D – Graves' tissue; H – FTC; K+L – PTC carcinomas). There was no relationship between pendrin expression or pendrin localisation and tumour stages (TNM), or tumour size (Table 2). The analysed anaplastic carcinomas with characteristics of the fully dedifferentiated tumours and no expression of thyroid-specific genes as well as the few cases of medullary carcinomas derived from C cells of neuronal crest origin were negative for PDS gene transcript and pendrin protein in IHC and Western blots.

## DISCUSSION

The iodide transporting properties of pendrin in follicular cells of the thyroid were suggested by experimental data on nonpolarised cell systems (Royaux *et al*, 2000; Yoshida *et al*, 2002), and demonstrated very recently on a polarised cell system (Gillam *et al*, 2004). The latter study provided evidence for pendrin-mediated apical iodide efflux from polarised mammalian cells loaded with iodine (Gillam *et al*, 2004). Thus, the pendrin expression in pathological thyroid tissues, particularly in cancerous tissues, is of interest due to its iodide translocating activity, which serves as one of the iodide suppliers for organification processes. Using molecular and immunological methods, such as quantitative RT–PCR, immunochistochemistry and Western blot, we have clearly proved pendrin protein expression in a wide spectrum of both normal and pathological tissues, mainly tumours.

Using the qualitative RT–PCR method, pendrin mRNA was detected in 92% of thyroid carcinomas, including well-differentiated papillary and follicular carcinomas. However, the PDS gene expression indicated high variability. We found only slightly altered or even equal mRNA expression in 19% of cancer samples as compared with normal paired tissues. However, in the majority (51%) of tumours, a reduction of mRNA levels by a factor of 1.5–10 was observed, which is consistent with the results reported by Porra *et al*, (2002). Pendred syndrome gene expression was considerably lower in 23% of tumours. In some previous studies it was found that the PDS transcript content in differentiated thyroid tumours was decreased by a factor 2–1000, when compared to normal thyroid tissues (Bidart *et al*, 2000; Mian *et al*, 2001). In these studies, the immunochemical examination also revealed a variability of the pendrin protein expression. However, in a large proportion of the tumours, a heterogeneous weak staining was observed. Also, Western blot assay showed individual variability between samples, and the protein expression level was closely associated with the relative mRNA expression as estimated by quantitative real-time PCR. Pendrin protein immunorecativity was found in all thyroid cancer tissues with MNE above 0.2. Using a similar method, Porra *et al* (2002) observed that protein levels corroborated RT–PCR data in the majority of PTSc. Pendrin was clearly demonstrated by means of immunohistochemic method in 73.3% of FTCs and 76.7% of PTCs with overall intracellular localisation. The results of the Western blot and IHC as comparative methods were complementary.

Our findings, which confirm to previous findings on the high variability of the PDS transcript level in differentiated thyroid carcinomas, were different from those of some previous studies (Bidart *et al*, 2000; Mian *et al*, 2001; Kondo *et al*, 2003). The differences can be explained by the use of different antibodies. In our study, we used high-affinity antigen-purified antibodies against amino- and carboxyl-termini of the pendrin molecule both located intracellularly (Gillam *et al*, 2004). Both antibodies displayed an identical pattern of reactivity in IHC and Western blot. The same patterns of pendrin distribution in thyroid carcinoma cells was observed using seven different anti-pendrin antibodies produced by us (data not shown). The immunohistochemical study reflects previous findings which show that pendrin is located at the apical pole of the follicular cells (Bidart *et al*, 2000; Royaux *et al*, 2000; Porra *et al*, 2002). This localisation is in agreement with the localisation of key proteins involved in the processes of thyroid hormone biosynthesis to which pendrin is thought to contribute (Royaux *et al*, 2000; Taurog, 2000; Yoshida *et al*, 2002). In the normal thyroid, only parts of the columnar follicular cells, which are considered to be functionally active, were stained. In NG and GD thyroids, the majority of follicular cells were intensely stained, especially in the small and proliferative follicles. This suggests that pendrin expression may be related to the functional status of the follicle and that of the single follicular cell. The major observation in our immunohistochemical analysis, however, is that in almost all (>70%) differentiated thyroid tumours, both follicular and papillary thyroid carcinomas, which preserved pendrin expression, the protein was present exclusively in the intracellular compartment. It should be noted that apical staining for pendrin was detected in normal thyroid tissues adjacent to the tumour analysed. The peptide immunoprecipitation tests resulted also in negative immunolabelling, which excludes technical pitfalls during immunostaining procedures. Gerard *et al* (2003) also reported an impairment of pendrin protein expression in pathological thyroids. They detected pendrin only in follicular carcinomas, at a level lower than that of normal thyroids. In our series of carcinomas of both histotypes, PTCs and FTCs, the incidence of pendrin-positive IHC was almost identical. We also analysed the relationship between pendrin protein expression levels and tumour size and tumour stage. However, no association was detected.

In follicular adenomas, a shift of pendrin localisation from the apical membrane to the cytoplasm was observed suggesting partial retention of protein in intracellular compartments, most likely membrane-associated. Although individual heterogeneity in the expression of pendrin was observed, all tissues expressing simultaneously a cytoplasmic and membranous localisation of pendrin had characteristics of cold adenomas.

Active translocation of iodide requires properly targeted pendrin at the apical plasma membrane (Taylor *et al*, 2002; Gillam *et al*, 2004). Therefore, the observation that in the majority of the analysed differentiated thyroid carcinomas pendrin remains in an intracellular membrane compartment may suggest that in these tissues pendrin is nonfunctional. Since improper targeting of pendrin was observed in almost all differentiated thyroid carcinomas and since pendrin mRNA and protein expression were comparable to noncancerous thyroid tissues, one may speculate that this is a general phenomenon of trafficking of pendrin in thyroid neoplasm. The causes responsible for alterations of the intracellular distribution of pendrin remain unknown. Several mechanisms could account for intracellular retention of pendrin in thyroid tumours cells. For example, changes in proper folding and assembly of protein might be considered. However, the methods we applied cannot give any information on how far the cytoplasmic pendrin is altered by sequence, conformation and function or if cofactors for trafficking the molecule are affected. Further studies are necessary to elucidate the detailed mechanism/s by which this protein expression is regulated in thyroid differentiated carcinoma cells and may contribute to the carcinogenesis and the development of these tumours. Investigations in this respect go beyond the possibilities presented by tissues specimens obtained from surgery.

## Figures and Tables

**Table 1:** Immunohistochemical results of pendrin staining

	**Staining grade**								
		**Intense**			**Moderate**				
**Tissues**	**No. of cases**	**m^a^**	**m+c**	**c**	**m**	**m+c**	**c**	**Focal**	**No staining**
Normal thyroid	47	47							
Graves' disease	10	10							
Nodular goiter	28	28							
Follicular adenoma	15	1	5	3		5	1		
Follicular carcinoma	15		1	6			4	1	3
Papillary carcinoma	30			19			4	3	4
Anaplastic carcinoma	2								2
Medullary carcinoma	8								8

a m=membranous staining; m+c=membranous and cytoplasmic staining; c=cytoplasmic staining.

**Table 2:** Pendrin expression and localisation in relation to tumour size and TNM (tumour-node-metastasis) in analysed thyroid neoplasm

**Patient**	**Sex/age**	**Histology**	**Tumour size (cm)**	**TNM**	**Pendrin immunostaining and localisation**
BW	F/66	PTC	1.3	T2aNo	+++; c
SJ	M/59	PTC	0.7	T1aNo	++; c
CH M	F/72	PTC	5 × 5 × 4	T3a	+++; c
UB	F/39	PTC	2 × 1 × 1	T2a	+++; c
LZ	F/72	PTC	2.2	T2a	+++; c
AA	F/28	PTC	1.1	T2a	++; c
GW	M/54	PTC	1.2	T2aNo	+++; c
KA	F/36	PTC	6; 2	T3b	+++; c
KK	F/23	PTC	1.5; 2	T2bNo	+++; c
MM	F/42	PTC	0.9 × 0.6	T1a	+++; c
SM	F/54	PTC	1.4	T2aNo	0
ZJ	F/70	PTC	1.8 × 1.1 × 1	T2aNo	++; c
KZ	F/63	PTC	1 × 1; 1 × 0.8	T1bNo	+++; c
GJ	F/53	PTC	4; 1	T2bNo	++; c
SG	F/49	PTC	2.5 × 2 × 2	T2aNo	0
TP	F/45	PTC	1.1; 1.5	T2bNo	++; c
GU	F/31	PTC	1.6	T2aNo	+++; c
SB	F/49	PTC	1.6 × 1.2	T2aNo	++; c
DG	F/38	PTC	1	T1aNo	++; c
KJ	F/41	PTC	1.1	T2aNo	+++; c
ZM	F/21	PTC	2.5	T2aN1a	0
GJ	F/51	PTC	2	T2aNo	+++; c
WB	F/22	PTC	5 × 6 × 6	T3aN1b	0
CA	F/37	PTC	1.1 × 1.2	T2aNo	+++; c
LA	F/32	PTC	1.4	T2aNo	+++; c
FJ	F/47	PTC	1.8	T2aNo	+++; c
BW	F/31	PTC	2.2	T2aNo	+++; c
KP	F/27	PTC	1.3	T2aNo	+++; c
WJ	F/53	PTC	1.2	T2aNo	+++; c
PM	F/38	PTC	1.7	T2aNo	+++; c
					
PT	F/44	FTC	6	T3	++; c
UB	M/65	FTC	4.8 × 3.6	T3	0
BJ	F/45	FTC	3	T2	+++; c
NE	F/65	FTC	3.5	T2	+++; c
FE	F/23	FTC	3	T2	++; c
SL	M/44	FTC	3.5	T2	+++; c
MB	F/38	FTC	1.4	T2	+++; c
WM	F/67	FTC	2.8 × 2.5	T2	0
SA	F/30	FTC	1.6	T2	++; c
WS	F/58	FTC	2.8	T2	+++; c
GM	F/53	FTC	1.3	T2	++; c
DS	F/47	FTC	1.4 × 1.8	T2	++; c
PJ	F/81	FTC	2.2 × 1.6	T2	+++; c+m
PA	F/18	FTC	6	T2	+++; c
ZZ	F/52	FTC	5.4	T3	0
					
DE	F/33	F. adenoma	3		+++; c+m
TR	F/57	F. adenoma	5 × 3		++; c+m
SE	F/54	F. adenoma	2.8 × 2.2		++; c+m
SB	F/48	F. adenoma	6		++; c+m
DG	F/44	F. adenoma	2.5 × 3.5		++; c+m
LM	F/49	F. adenoma	2		++; c
UA	F/64	F. adenoma	2		+++; c
SJ	F/39	F. adenoma	1.2		++; c
JM	F/43	F. adenoma	2.8 × 1.9		+++; c
SB	F/60	F. adenoma	2		+++; c+m
CA	F/46	F. adenoma	2.3		++; c
BJ	F/37	F. adenoma	1.5		+++; c+m
JJ	F/76	F. adenoma	3.2		+++; c
WM	F/33	F. adenoma	5		+++; m
OB	F/31	F. adenoma	3		+++; c

Intensity score: 0=null; ++=moderate; +++=intense; c=cytoplasmic; m=membranous; F=focal.
